# Effect of Lorazepam on the Development of the Hairy Maggot Blow Fly, *Chrysomya rufifacies* (Macquart): Implication for Forensic Entomology

**DOI:** 10.1155/2023/1051736

**Published:** 2023-07-10

**Authors:** Sarika Annasaheb Bansode, Vitthal Ramrao More

**Affiliations:** ^1^Department of Zoology, Kelkar Education Trust's V. G. Vaze College of Arts, Science and Commerce, Mithagar Road Mulund (East), Mumbai 400081, India; ^2^Department of Zoology, Government College of Arts and Science, Aurangabad 431004, Maharashtra, India

## Abstract

Entomotoxicology is based on using insect evidence recovered from a dead body to find out the cause and time of the death. Drugs can accumulate in fly larvae when they ingest the flesh of deceased persons and alter the normal development of the fly causing implications in calculating postmortem intervals. Lorazepam is an antidepressant generally used to treat anxiety. Larvae of *Chrysomya rufifacies* were fed on the beef liver mixed with lorazepam to study the effect of lorazepam on the developmental rate of larvae and to count delay in postmortem interval. Larvae grown on the beef liver with different doses of lorazepam showed delayed development as compared to normal larvae. The life cycle durations in experimental cultures with different concentrations of lorazepam completed in 1 ppm (272.56 hrs), 2 ppm (289.23 hrs), 3 ppm (324.10 hrs), and 4 ppm (350.72 hrs), while in the control culture life cycle completed in 257.26 hrs. The length, weight, and width of the larvae treated with lorazepam were smaller than the untreated culture. Length, weight, and width decreased with increased concentration of lorazepam. This delay in development ultimately affects the postmortem interval. That is why prior knowledge of the life cycle of flies with respect to various drugs needs to be studied, and these baseline data can be used to calculate postmortem interval and cause of death.

## 1. Introduction

Insect's life cycle acts as a precise clock that begins soon after death. That is why insects are called the first entity to witness death and are used to calculate postmortem intervals. Forensic entomology makes use of insects and insect evidence to help law enforcement to determine the accurate time and cause of the death [1]. When using entomological evidence, two considerations are most important. First, insects often lay eggs within a few minutes or hours of death. That is why the cycle of development of the oldest maggots feeding on the corpse shows the most approximate time since death [2]. Second, the pattern of insect succession is also a good indicator of PMI because insects arrive at predictable and successive waves based on stages of decomposition [3, 4].

Generally, Calliphoridae flies colonize first on a cadaver because they have the ability to travel over a 15 km distance after getting attracted by the odor produced during decomposition. Females start to oviposit within the first few hours after death [5]. When traditional specimens such as blood, urine, or muscle tissues are unavailable, insects and insect remnants can also be used for toxicological examinations [6–8]. Entomotoxicology is another important and interesting area to investigate the effects of drugs and toxins on arthropod development [9]. The pattern of cadaveric changes in soft tissue structures tends to indicate how long a person has been dead. However, changing the breakdown of organic matter can significantly alter the estimated time of death [10]. When dipteran larvae feed on intoxicated tissue they in turn metabolize the substance and incorporate it into their own tissue. Such larvae can also be useful in the toxicological analysis as secondary bioaccumulation occurs in the larval body; it can tell us which toxin was present in the body. During different experimental studies, a large number of poisonous chemicals were recovered from maggots that feed upon animals, which died due to the intake of certain chemicals. Because of the rise in drug-related deaths, it is essential to know how all these drugs influence the development of flies that feed on cadavers in order to prevent mistakes in PMI estimates [11].

In the present study, the effect of lorazepam on the development of forensic fly *C. rufifacies* is studied. These baseline data are valuable as it can contribute to the forensic entomologists to calculate accurate PMI. Knowledge of the drug-specific life cycle of a fly saves time and takes an entomologist a direct conclusion to calculate PMI. Many times, it is observed that the suicidal or murderer victims have been given sedative drugs and under such conditions, it is found that these drugs accumulate in larval body tissue and these drugs can affect the duration of the life cycle stages. In such conditions, it becomes hard to find correct postmortem intervals (PMI). That is why it is necessary to have the standard data related to such drugs and their effects on the duration of the life cycle stages and the impact on their morph metric measurement.

## 2. Methodology

### 2.1. Lorazepam

Lorazepam is an antianxiety agent belonging to the class benzodiazepine. The FDA approves it for short-term relief of anxiety symptoms such as anxiety disorders, anxiety-associated insomnia, anesthesia premedication in adults to relieve anxiety or to produce amnesia, and treatment of status epilepticus [12]. Overdose of lorazepam may cause CNS and respiratory depression. It also leads to hypotension, ataxia, confusion, and coma and can be fatal. Each tablet contains 0.5 mg, 1 mg, or 2 mg of lorazepam [13].

### 2.2. Collection of Sample

Sample collection was carried out in the Osmanabad district of Maharashtra state, India. The fresh meat was purchased from the local slaughterhouse and allowed to be partially putrefied. Partially putrefied meat was exposed to the air and the flies were attracted to it within a few minutes. Flies were collected with the help of an insect net. Similarly, maggots and adult flies were also collected from different road cadavers of different animals. Flies were collected with the help of an insect net and larvae were collected with the help of forceps and kept in the 500 ml beaker. Collected flies and larvae were brought to the laboratory and reared in laboratory conditions.

### 2.3. Laboratory Rearing

Adults were fed daily on fresh liver and honey mixed in water. The fresh liver was used as an oviposition site for females. Fresh liver and honey water were provided in a separate Petri dish. Hygiene and cleanliness were maintained in the rearing boxes to avoid any infection of the culture. Daily Petri dishes were cleaned and dried to avoid infection of the culture [14].

Cultures of the flies were kept under continuous observation and after each hour it was checked to ensure egg laying. In the beginning, all flies were kept in a cage but to maintain species-specific culture proper identification was important. As soon as the fly laid eggs, eggs were separated and kept in the separate cage to maintain the pure culture of each isofemale. Temperature and humidity data were recorded throughout the experiment. The first, second, and third instar were dissected to ensure correct species identification based on morphological characters. Adult flies are also examined to ensure correct identification using previously published standard identification keys [15, 16]. After morphology-based identification, DNA barcoding of mtDNA using cytochrome oxidase subunit I was performed to confirm species identification. The sequence was uploaded to the BOLD to confirm the identification of molecular analysis of the data. The GenBank accession number of *C. rufifacies* is (MG816778) [17]. To measure morphological parameters larvae were immersed in hot water to aid the straightening which helped in proper measurement.

### 2.4. Experimental Setup

Larvae of *C. rufifacies* were collected from the pure culture maintained at the laboratory. 500 gm of the fresh beef liver was purchased from the slaughterhouse and it was finely chopped. Lorazepam 1 mg was used for the present study. The concentrations of lorazepam were set as 1 ppm, 2 ppm, 3 ppm, and 4 ppm, respectively. One set was maintained as a control free from lorazepam doses. 20 larvae were introduced to each set and development was studied till the emergence of an adult. The duration required for the development of each stage in different concentrations was recorded with respect to temperature and humidity. A total of four replicates were taken of the present research. Temperature and humidity records are presented in the form of graphs.

### 2.5. Statistical Analysis

A statistical analysis is performed using SPSS. One-way ANOVA with post hoc test was performed for comparison.

## 3. Results

The developmental time of *C. rufifacies* in treated cultures with different concentrations of lorazepam was completed in 272.56 hrs (1 ppm), 289.23 hrs (2 ppm), 324.10 hrs (3 ppm), and 350.72 hrs (4 ppm), respectively, while in the control culture, the life cycle was completed in 257.26 hrs. Mean values for recorded temperature varied from 23.08 ± 6.940°C for 4 ppm dose to 25.36 ± 6.030°C for the 1 ppm dose, while mean values of relative humidity ranged from 45.73 ± 8.957°C for 4 ppm dose to 56.63 ± 6.834°C for 2 ppm (Table 1).

**Figure 1:**
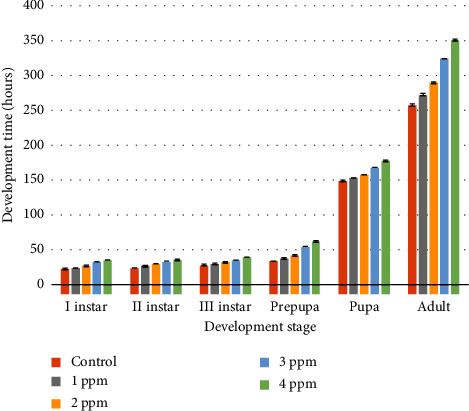
Graphical representation of the developmental time of different stages of *C. rufifacies* in the treated and untreated cultures. The time required to complete the development is higher in treated cultures than in untreated cultures and it is delayed with the increased concentration of lorazepam. Colours below the bars indicate the results of the Tukey comparison, where the same colours did not differ statistically to *α* = 0.05.

As compared to the control culture there was a significant difference in the life cycle of the treated culture and the life cycle was delayed in 1 ppm, 2 ppm, 3 ppm, and 4 ppm by 15.3, 31.97, 66.84, and 93.46 hrs, respectively (Figure 1). This difference in life cycle duration affecting the PMI is the reason why prior knowledge of the insect's dataset is fundamental. The feeding stages in the control spent 33.48 hrs while in the treated cultures with lorazepam 1 ppm, 2 ppm, 3 ppm, and 4 ppm spent 37.70 hrs, 42.21 hrs, 54.57 hrs, and 62.36 hrs, respectively. The postfeeding and pupa stages in control spent 148.70 hrs and in the treated cultures with different doses of lorazepam 1 ppm, 2 ppm, 3 ppm, and 4 ppm spent 153.63, 157.66, 168.02, and 177.80 hrs, respectively (Figure 1). Multiple comparisons done by using a post hoc test showed that there were a highly significant differences between each developmental stage in the lorazepam-treated culture and the control culture. Developmental time varied significantly in all treated cultures. Details of data of the post hoc test and results can be found in supplementary files (https://osf.io/zd269/?view_only=d3081187a13040d5b04c26048901f1c4). Figure 1 shows the graphical representation of the developmental time of different stages of *C. rufifacies* in treated and untreated cultures. Whereas Table 2 shows the developmental time parameters of different stages of *C. rufifacies* in treated and untreated cultures. The time required to complete the development is higher in treated cultures than in untreated cultures and it is delayed with the increased concentration of lorazepam. The life cycle of the fly was disturbed due to the increase in the concentration of lorazepam. With the increases in doses of lorazepam, the larval development was slowed and the pupal development was also delayed. In the control set development was normal and flies emerged first from it. In treated cultures, the life cycle duration increased with the increase in concentrations of lorazepam.

Morphometric parameters such as weight, length, and width of different stages in control were slightly bigger than in the treated cultures, even in treated cultures sizes varied depending on the concentration of the drug in the cultures. The sizes of different stages decreased with an increase in the concentration of lorazepam. Larvae in high concentration were smaller in length, weight, and width than the larvae in low concentration.

The morphometric parameters of length (Table 3) for *C. rufifacies* in untreated cultures was I instar (4.2 ± 0.081 mm), II instar (8.1 ± 0.081 mm), III instar (16.3 ± 0.081 mm), prepupa (13.5 ± 0.081 mm), pupa (10.42 ± 0.095 mm), and adult (10.27 ± 0.07 mm); in 1 ppm: I instar (3.97 ± 0.095 mm), II instar (7.1 ± 0.081 mm), III instar (14.37 ± 0.05 mm), prepupa (11.57 ± 0.08 mm), pupa (9.42 ± 0.095 mm), and adult (9.07 ± 0.095 mm); in 2 ppm: I instar (3.8 ± 0.081 mm), II instar (6.12 ± 0.095 mm), III instar (13.12 ± 0.095 mm), prepupa (10.5 ± 0.081 mm), pupa (9.05 ± 0.057 mm), and adult (8.5 ± 0.081 mm); whereas in 3 ppm: I instar (3.35 ± 0.057 mm), II instar (5.72 ± 0.095 mm), III instar (11.02 ± 0.05 mm), prepupa (13.5 ± 0.081 mm), pupa (8.45 ± 0.05 mm), and adult (8.07 ± 0.05 mm); and in 4 ppm: I instar (3.05 ± 0.05 mm), II instar (5.07 ± 0.095 mm), III instar (10.02 ± 0.05 mm), prepupa (8.87 ± 0.05 mm), pupa (7.47 ± 0.05 mm), and adult (7.17 ± 0.05 mm), respectively.

The width of *C. rufifacies* (Table 4) in untreated cultures was I instar (1.47 ± 0.09 mm), II instar (1.85 ± 0.08 mm), III instar (3.57 ± 0.05 mm), prepupa (3.58 ± 0.07 mm), pupa (3.37 ± 0.09 mm), and adult (3.30 ± 0.08 mm); in 1 ppm: I instar (1.35 ± 0.05 mm), II instar (1.57 ± 0.02 mm), III instar (3.42 ± 0.08 mm), prepupa (3.23 ± 0.08 mm), pupa (3.1 ± 0.05 mm), and adult (2.8 ± 0.02 mm); in 2 ppm: I instar (1.57 ± 0.07 mm), II instar (1.57 ± 0.07 mm), III instar (1.97 ± 0.097 mm), prepupa (2.86 ± 0.017 mm), pupa (2.58 ± 0.09 mm), and adult (2.72 ± 0.07 mm); whereas in 3 ppm: I instar (0.9 ± 0.04 mm), II instar (1.36 ± 0.09 mm), III instar (1.36 ± 0.09 mm), prepupa (2.55 ± 0.05 mm), pupa (2.58 ± 0.04 mm), and adult (2.45 ± 0.096 mm); and in 4 ppm: I instar (0.8 ± 0.19 mm), II instar (1.28 ± 0.07 mm), III instar (2.05 ± 0.05 mm), prepupa (2.44 ± 0.03 mm), pupa (2.33 ± 0.19 mm), and adult (2.16 ± 0.016 mm), respectively.

The weight of *C. rufifacies* (Table 5) in untreated cultures was I instar (9.45 ± 0.05 mg), II instar (19.15 ± 0.12 mg), III instar (51.25 ± 0.17 mg), prepupa (46.77 ± 0.18 mg), pupa (4.71 ± 0.012 mg), and adult (37.55 ± 0.01 mg); in 1 ppm: I instar (9.12 ± 0.15 mg), II instar (16.20 ± 0.14 mg), III instar (46.55 ± 0.05 mg), prepupa (41.85 ± 0.005 mg), pupa (34.41 ± 0.009 mg), and adult (30.38 ± 0.08 mg); in 2 ppm: I instar (8.6 ± 0.11 mg), II instar (14.57 ± 0.09 mg), III instar (43.12 ± 0.15 mg), prepupa (40.67 ± 0.20 mg), pupa (31.89 ± 0.02 mg), and adult (26.62 ± 0.09 mg); whereas in 3 ppm: I instar (8.47 ± 0.005 mg), II instar (13.07 ± 0.09 mg), III instar (40.07 ± 0.09 mg), prepupa (35.53 ± 0.05 mg), pupa (28.41 ± 0.017 mg), and adult (23.17 ± 0.12 mg); and in 4 ppm: I instar (8.15 ± 0.12 mg), II instar (12.1 ± 0.14 mg), III instar (36.45 ± 0.12 mg), prepupa (33.32 ± 0.23 mg), pupa (26.62 ± 0.15 mg), and adult (20.5 ± 0.08 mg), respectively.

**Figure 2:**
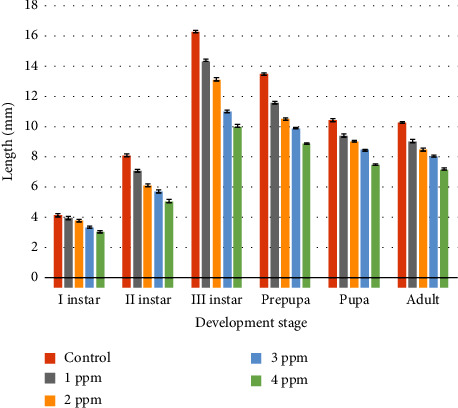
Graphical representation of means of the length of different development stages of *C. rufifacies* in treated and untreated cultures. Observed length in untreated culture is higher than in treated cultures and it decreases with the increased concentration of lorazepam. Least length recorded in culture with a high dose of lorazepam. Colours below the bars indicate the results of the Tukey comparison, where the same colours did not differ statistically to *α* = 0.05.

**Figure 3:**
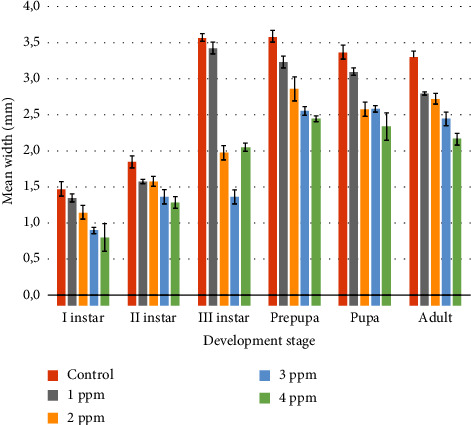
Graphical representation of means of the width of different development stages of *C. rufifacies* in treated and untreated cultures. Observed width in untreated culture is higher than in treated cultures and it decreases with the increased concentration of lorazepam. The least width was recorded in culture with a high dose of lorazepam. Colours below the bars indicate the results of the Tukey comparison, where the same colours did not differ statistically to *α* = 0.05.

**Figure 4:**
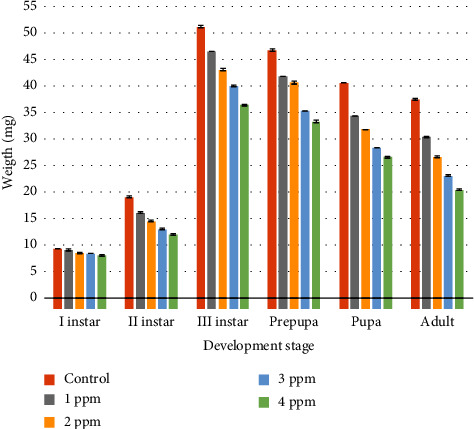
Graphical representation of means of the weight of different development stages of *C. rufifacies* in treated and untreated cultures. Observed weight in untreated cultures is higher than in treated cultures and it decreases with the increased concentration of lorazepam. Least weight recorded in culture with a high dose of lorazepam. Colours below the bars indicate the results of the Tukey comparison, where the same colours did not differ statistically to *α* = 0.05.

Results of ANOVA showed that there was a significant difference in each developmental stage of larvae when the control group compared with the treated group (1 ppm, 2 ppm, 3 ppm, and 4 ppm). In the control set, 1 ppm, and 2 ppm no mortality was observed and the emergence of the adult flies was 100% but in treated cultures of 3 ppm and 4 ppm emergence was 50% only and adults were very small in size as compared to the control set. Detailed data regarding the variation in the developmental time and morphological parameters in the treated and untreated cultures can be found here. Figures 2–4 show the graphical representation of means of the length, width, and weight of different development stages of *C. rufifacies* in treated and untreated cultures. Observed length, width, and weight in untreated cultures are higher than in treated cultures and it decreases with the increased concentration of lorazepam. The least length, width, and weight were recorded in culture with a high dose of lorazepam. Colours below the bars indicate the results of the Tukey comparison, where the same colours did not differ statistically to *α* = 0.05.

Larvae in all treated cultures showed very fast and random movement. Most of the time they were trying to move away from the food and gather at the edges of the beaker. This movement was faster in the cultures with high concentrations of the drug as compared to the least concentration and controlled culture. Larvae in the untreated cultures were voracious feeders in feeding stages, whereas the rate of feeding was decreased in the cultures treated with lorazepam. Larvae treated in 3 ppm and 4 ppm having high doses of lorazepam showed decreased feeding than that to control, 1 ppm, and 2 ppm treated cultures.

## 4. Discussion

In the present research, the effect of lorazepam on the developmental duration and morphological parameters is performed. Beyer et al. [6] first proposed the use of insects in a toxicological analysis and since then many researchers have been using insects as evidence to determine the cause of death. Carrion flies, especially dipteran flies Calliphoridae and Sarcophagidae are extensively used in toxicological analysis. Insects have been demonstrated to be an effective alternative technique for toxicological analysis, especially when the dead body is only a skeleton. Drugs and toxins can be detected in larvae whenever their metabolic accumulation rate exceeds the excretion rate and alter the development of the fly life cycle. Alterations in the life cycle of the fly should be considered to calculate PMI accurately (de Carvalho) [18]. Goff and Lord [9] and Kintz et al. [19, 20] have demonstrated the detection of the prescription of drugs through the analysis of fly larvae feeding upon human remains. Toxicological tests were performed on the remains of a male decedent having a known postmortem interval of 67 days. Liquid chromatography was employed in the analysis of heart, liver, lungs, spleen, and kidney tissues as well as Calliphoridae fly larvae collected from the victim. Results of this analysis revealed the presence of five drugs namely triazolam, oxazepam, phenobarbital, alimemazine, and clomipramine in both the tissues and fly larvae examined. All drugs were present in aforementioned tissues except trizolam. Trizolam is not detected in either the spleen or the kidney samples. All five drugs were isolated from the developing fly larvae. It was not possible here to establish any quantitative correlations between the concentrations of the drugs detected in the fly larvae and the drugs present in human tissues.

Dayananda [21] have studied the effect of morphine and heroin on the development of flies. Morphine and heroin both slow down the rate of fly development. As per the examination, heroin has an effect on the development of flies and it actually speeds up larval growth and development. Then, it decreases the developmental rate of the pupal stage. This leads to an increase in the overall timing of development from an egg to an adult. Some effects of toxins on arthropods depend on the concentration of the toxin while others simply depend on the presence of the toxin. For example, the lethal dose of cocaine causes larvae to “develop more rapidly in between 36 and 76 hours after hatching.” The amount of growth and development depends on the concentration of cocaine in the area being fed. The amount of methamphetamine, on the other hand, affects the rate of pupal development. Similar results are obtained by Carvalho et al. [22], who studied the effect of diazepam on larvae of *C. albiceps* and *C. putoria* of the Calliphoridae family. The time required for pupariation and adult emergence was significantly greater than the control set of larvae. Similarly, morphological parameters (length, weight, and width) were also affected in treated cultures.

Kharbouche et.al. [23] studied the presence of codeine accumulation and elimination in larvae, pupae, and imago of the blowfly *Lucilia sericata* and its effect on its development. Results showed a 29 hrs interval bias on the evaluation of the larval stage duration calculated from the larvae weight that has to be considered if codeine was present in the larvae substrate. Similarly, a 21 hrs interval bias on the total duration of development, from egg to imago, has to be considered if codeine was present in the larvae substrate. Kanesarajah and Turner [10] have also shown the larval growth of the blowfly, *Calliphora vicina* was significantly faster by as much as 2 days on lung, kidney, heart, or brain tissues as compared to liver tissues which significantly causes the postmortem interval in a forensic cases.

A similar study performed by Andrade-Herrera et al.[24], they have also studied the effect of lorazepam on the development of larvae of *Calliphora vicina* and *Calliphora loewi*. Their findings showed that larvae feeding on drug-containing tissues in lower concentrations developed more rapidly and the emergence of adults was greater but with the increase in concentration developmental rate was delayed. Pupation was normal for the control group but it was significantly greater for the culture which fed on lorazepam dosed diet. Results emphasizing the importance of determining the contamination rates of lorazepam are essential to prevent deviation from PMI estimations.

Results obtained by Al Shuraym et al. [25] are similar to this research. They have studied the effect of zolpidem contaminated tissues on the dipteran flies *Chrysomya megacephala* (Fabricius 1794) and *Chrysomya saffranea* (Bigot 1877). As per the findings, the life cycle of *C. megacephala* in treated cultures of zolpidem tartrate (1, 2, 3, and 4 ppm) was completed in 266.17, 298.00, 318.97, and 346.23 h., whereas in the untreated culture, development took place in lesser time (244.55 h). The life cycle of *C. saffranea* in treated cultures of zolpidem tartrate (1, 2, 3, and 4 ppm) was completed in 255.00, 271.00, 290.33, and 310.33 h, whereas in the untreated culture, development took place in lesser time (223.93 h). Morphological parameters such as weight, length, and width of *C. megacephala* decreased with the increased concentration of zolpidem tartrate. Morphological parameters of *C. saffranea* showed a negative association with increased concentration of zolpidem tartrate. Somehow larval development did not show much variation in 1 ppm and 2 ppm due to the least concentration but as concentration increased the growth pattern was disturbed. While calculating the PMI concentration of drugs also needs to be considered for the correct estimation of time since death. A similar study done by Goff [26] has proved effects of cocaine and benzoylecognine on growth performance of larvae of *Boettcherisca peregrina* (Robineau-Desvoidy). Through intravenous injection, the rabbits were given 35, 69, and 137 mg of cocaine. Larvae developed faster on tissue containing cocaine, benzoylecognine, or both from rabbits injected with 69 and 137 mg of cocaine than on tissue from rabbits injected with 35 mg of cocaine or no cocaine from hours 30 to 70. Total development times for pupation and adult eclosion were reduced accordingly. These differences observed in the rate of development alter postmortem interval and creates inconvenience to the forensic entomologist.

Studies done by Bansode et al. [27, 28] showed that temperature plays a vital role in insect growth and development. Lower temperature and high humidity increase the life cycle duration whereas high temperature and low moisture fasten the developmental rate. While calculating the PMI, consideration of temperature data is very important for an entomologist to draw proper conclusions. In the present study, 32.93°C was the highest maximum recorded temperature and 64.88% was the recorded humidity when the fly took 324.10 hrs to complete its development. Similarly, 14.95 was the minimum recorded temperature and 35.23% was the minimum recorded humidity when the fly took 350.72 hrs to complete its development. Similar results are obtained by Bansode et al. [27] whose study showed that *Sarcophaga ruficornis* took 21 days to complete its life cycle when the temperature was 20°C, 18 days at 25°C, 14 days at 30°C, 11 days at 35°C, and 11 days at 40°C. Another study done by Bansode et al. [14, 28] shows that the total life cycle of *Lucilia cuprina* in the summer season was completed in 218 hrs. (9.08 days) when the average temperature was 31.6°C and average humidity was 26%, in the rainy season completed in 301 hrs. (12.54 days) when the average temperature was 29.2°C and average humidity was 67%, while in the winter season was completed in 274 hrs. (11.4 days) when the average temperature and humidity were 24.1°C and 39% respectively. Similarly, *Chrysomya megacephala* took 15 days to complete its life cycle when the temperature was 20°C, 12 days at 25°C, 10 days at 30°C, 08 days at 35°C, and 07 days at 40°C.

## 5. Conclusion

Insects can prove to be a valuable tool in the investigation of homicides, suicides, and other unattended death. Drugs and toxic chemicals present in a dead body accumulating in the larval body can affect its development and morphological parameters. Toxicological analysis of carrion insects can reveal the cause of death and also helps in calculating postmortem intervals. Carrion flies may give different responses to different drugs and chemicals. Lorazepam has shown a significant effect on the development and morphological parameters; hence, it is important to take this into consideration while calculating postmortem interval.

## Figures and Tables

**Table 1:** Mean values of temperature and relative humidity were recorded by hygrothermometer while evaluating the effect of different doses of lorazepam on the development of the hairy maggot blow fly, *Chrysomya rufifacies* (Macquart).

Variable	Dose	Mean	Maximum	Minimum	Std. deviation	Std. error
Temperature (°C)	Control	25.10	31.73	18.48	5.695	2.014
	1 ppm	25.36	32.43	18.30	6.030	2.132
	2 ppm	25.33	32.25	18.40	5.911	2.090
	3 ppm	25.28	32.93	17.63	6.547	2.315
	4 ppm	23.08	31.20	14.95	6.940	2.454

Humidity (%)	Control	52.78	60.05	45.50	6.233	2.204
	1 ppm	55.31	61.00	49.63	4.926	1.742
	2 ppm	56.63	64.63	48.63	6.834	2.416
	3 ppm	56.61	64.88	48.35	7.061	2.496
	4 ppm	45.73	56.23	35.23	8.957	3.167

**Table 2:** Developmental time parameters of different stages of *C. rufifacies* in the treated and untreated cultures.

Dose	Dev stage	Mean	SD	SE	Min	95% CI		Max
						Low	Up	
Control	I instar	22.59	0.57	0.29	22.00	21.68	23.50	23.15
	II instar	24.31	0.53	0.26	24.00	23.47	25.16	25.10
	III instar	28.18	0.67	0.33	27.40	27.12	29.23	29.00
	Prepupa	33.49	0.36	0.18	33.20	32.92	34.06	34.00
	Pupa	148.70	0.67	0.33	148.10	147.64	149.76	149.50
	Adult	257.26	1.85	0.93	255.70	254.32	260.21	259.65

1 ppm	I instar	24.46	0.49	0.24	24.00	23.68	25.24	25.15
	II instar	26.86	0.45	0.22	26.20	26.15	27.57	27.15
	III instar	29.90	0.62	0.31	29.00	28.91	30.89	30.40
	Prepupa	37.70	0.41	0.20	37.30	37.05	38.35	38.10
	Pupa	153.64	0.48	0.24	153.15	152.87	154.40	154.10
	Adult	272.56	1.48	0.74	270.75	270.21	274.91	274.10

2 ppm	I instar	27.18	0.77	0.39	26.15	25.95	28.40	28.00
	II instar	29.96	0.38	0.19	29.40	29.36	30.56	30.20
	III instar	32.23	0.70	0.35	31.40	31.11	33.34	33.10
	Prepupa	42.21	0.71	0.35	41.30	41.09	43.33	43.00
	Pupa	157.66	0.56	0.28	157.00	156.77	158.55	158.15
	Adult	289.24	1.15	0.58	287.85	287.40	291.07	290.65

3 ppm	I instar	32.96	0.38	0.19	32.40	32.36	33.56	33.20
	II instar	33.61	0.51	0.25	33.15	32.81	34.42	34.10
	III instar	34.93	0.36	0.18	34.40	34.35	35.50	35.20
	Prepupa	54.58	0.40	0.20	54.25	53.94	55.21	55.15
	Pupa	168.03	0.46	0.23	167.40	167.30	168.75	168.50
	Adult	324.10	0.45	0.22	323.55	323.39	324.81	324.65

4 ppm	I instar	35.35	0.51	0.25	35.00	34.54	36.16	36.10
	II instar	35.61	0.51	0.25	35.15	34.81	36.42	36.10
	III instar	39.60	0.52	0.26	39.10	38.77	40.43	40.10
	Prepupa	62.36	0.43	0.21	62.10	61.68	63.04	63.00
	Pupa	177.80	0.47	0.23	177.10	177.05	178.55	178.10
	Adult	350.73	1.00	0.50	349.45	349.13	352.32	351.60

The time required to complete the development is higher in treated cultures than in untreated cultures and it is delayed with the increased concentration of lorazepam. Dev stage = development stage; SD = standard deviation; SE = standard error; min = minimum; CI = confidence interval; low = lower bound; up = upper bound; max = maximum.

**Table 3:** Length parameters of different stages of *C. rufifacies* in the treated and untreated cultures.

Dose	Dev stage	Mean	SD	SE	Min	95% CI		Max
						Low	Up	
Control	I instar	4.20	0.08	0.04	4.10	4.07	4.33	4.30
	II instar	8.10	0.08	0.04	8.00	7.97	8.23	8.20
	III instar	16.30	0.08	0.04	16.20	16.17	16.43	16.40
	Prepupa	13.50	0.08	0.04	13.40	13.37	13.63	13.60
	Pupa	10.43	0.10	0.05	10.30	10.27	10.58	10.50
	Adult	10.28	0.05	0.03	10.20	10.20	10.35	10.30

1 ppm	I instar	3.98	0.10	0.05	3.90	3.82	4.13	4.10
	II instar	7.10	0.08	0.04	7.00	6.97	7.23	7.20
	III instar	14.38	0.05	0.03	14.30	14.30	14.45	14.40
	Prepupa	11.58	0.09	0.04	11.50	11.44	11.71	11.65
	Pupa	9.43	0.10	0.05	9.30	9.27	9.58	9.50
	Adult	9.08	0.10	0.05	9.00	8.92	9.23	9.20

2 ppm	I instar	3.80	0.08	0.04	3.70	3.67	3.93	3.90
	II instar	6.13	0.10	0.05	6.00	5.97	6.28	6.20
	III instar	13.13	0.10	0.05	13.00	12.97	13.28	13.20
	Prepupa	10.50	0.08	0.04	10.40	10.37	10.63	10.60
	Pupa	9.05	0.06	0.03	9.00	8.96	9.14	9.10
	Adult	8.50	0.08	0.04	8.40	8.37	8.63	8.60

3 ppm	I Instar	3.35	0.06	0.03	3.30	3.26	3.44	3.40
	II instar	5.73	0.10	0.05	5.60	5.57	5.88	5.80
	III instar	11.03	0.05	0.03	11.00	10.95	11.10	11.10
	Prepupa	9.93	0.03	0.01	9.90	9.88	9.97	9.95
	Pupa	8.45	0.06	0.03	8.40	8.36	8.54	8.50
	Adult	8.08	0.05	0.03	8.00	8.00	8.15	8.10

4 ppm	I Instar	3.05	0.06	0.03	3.00	2.96	3.14	3.10
	II instar	5.08	0.10	0.05	5.00	4.92	5.23	5.20
	III instar	10.03	0.05	0.03	10.00	9.95	10.10	10.10
	Prepupa	8.88	0.05	0.03	8.80	8.80	8.95	8.90
	Pupa	7.48	0.05	0.03	7.40	7.40	7.55	7.50
	Adult	7.18	0.05	0.03	7.10	7.10	7.25	7.20

The length (mm) in treated cultures is less than in untreated cultures and it decreases with the increased concentration of lorazepam. Dev stage = development stage; SD = standard deviation; SE = standard error; min = minimum; CI = confidence interval; low = lower bound; up = upper bound; max = maximum.

**Table 4:** Width parameters of different stages of *C. rufifacies* in the treated and untreated cultures.

Dose	Dev stage	Mean	SD	SE	Min	95% CI		Max
						Low	Up	
Control	I instar	1.48	0.10	0.05	1.40	1.40	1.55	1.50
	II instar	1.85	0.09	0.04	1.80	1.76	1.94	1.90
	III instar	3.58	0.05	0.03	3.50	3.42	3.73	3.70
	Prepupa	3.59	0.08	0.04	3.50	3.45	3.72	3.70
	Pupa	3.38	0.10	0.05	3.30	3.30	3.45	3.40
	Adult	3.30	0.09	0.04	3.21	3.18	3.43	3.40

1 ppm	I instar	1.35	0.05	0.03	1.30	1.26	1.44	1.40
	II instar	1.58	0.02	0.01	1.50	1.42	1.73	1.70
	III instar	3.43	0.08	0.04	3.40	3.35	3.50	3.50
	Prepupa	3.23	0.08	0.04	3.20	3.19	3.27	3.25
	Pupa	3.10	0.05	0.03	3.00	2.97	3.23	3.20
	Adult	2.80	0.02	0.01	2.70	2.67	2.93	2.90

2 ppm	I instar	1.15	0.10	0.05	1.10	1.06	1.24	1.20
	II instar	1.58	0.08	0.04	1.50	1.42	1.73	1.70
	III instar	1.98	0.10	0.05	1.90	1.82	2.13	2.10
	Prepupa	2.86	0.17	0.09	2.80	2.74	2.98	2.95
	Pupa	2.58	0.10	0.05	2.50	2.42	2.74	2.70
	Adult	2.73	0.08	0.04	2.50	2.45	3.00	2.90

3 ppm	I instar	0.90	0.04	0.02	0.80	0.77	1.03	1.00
	II instar	1.37	0.10	0.05	1.32	1.30	1.43	1.40
	III instar	1.37	0.10	0.05	1.32	1.30	1.43	1.40
	Prepupa	2.56	0.06	0.03	2.50	2.40	2.71	2.70
	Pupa	2.58	0.04	0.02	2.50	2.43	2.73	2.71
	Adult	2.45	0.10	0.05	2.40	2.36	2.54	2.50

4 ppm	I instar	0.80	0.19	0.10	0.70	0.67	0.93	0.90
	II instar	1.29	0.08	0.04	1.22	1.22	1.35	1.31
	III instar	2.05	0.06	0.03	1.80	1.75	2.35	2.20
	Prepupa	2.45	0.04	0.02	2.35	2.32	2.57	2.52
	Pupa	2.34	0.19	0.10	2.30	2.25	2.43	2.42
	Adult	2.17	0.08	0.04	2.13	2.11	2.23	2.20

The width (mm) in treated cultures is less than that of the untreated cultures and it decreases with the increased concentration of lorazepam. Dev stage = development stage; SD = standard deviation; SE = standard error; min = minimum; CI = confidence interval; low = lower bound; up = upper bound; max = maximum.

**Table 5:** Weight parameters of different stages of *C. rufifacies* in the treated and untreated cultures.

Dose	Dev stage	Mean	SD	SE	Min	95% CI		Max
						Low	Up	
Control	I instar	9.45	0.06	0.03	1.40	1.40	1.55	1.50
	II instar	19.16	0.13	0.06	1.80	1.76	1.94	1.90
	III instar	51.25	0.17	0.09	3.50	3.42	3.73	3.70
	Prepupa	46.78	0.19	0.09	3.50	3.45	3.72	3.70
	Pupa	40.71	0.01	0.01	3.30	3.30	3.45	3.40
	Adult	37.55	0.10	0.05	3.21	3.18	3.43	3.40

1 ppm	I instar	9.13	0.15	0.08	1.30	1.26	1.44	1.40
	II instar	16.20	0.14	0.07	1.50	1.42	1.73	1.70
	III instar	46.55	0.06	0.03	3.40	3.35	3.50	3.50
	Prepupa	41.86	0.01	0.00	3.20	3.19	3.27	3.25
	Pupa	34.41	0.01	0.00	3.00	2.97	3.23	3.20
	Adult	30.38	0.09	0.04	2.70	2.67	2.93	2.90

2ppm	I instar	8.60	0.12	0.06	1.10	1.06	1.24	1.20
	II instar	14.58	0.10	0.05	1.50	1.42	1.73	1.70
	III instar	43.13	0.15	0.08	1.90	1.82	2.13	2.10
	Prepupa	40.68	0.21	0.10	2.80	2.74	2.98	2.95
	Pupa	31.89	0.03	0.01	2.50	2.42	2.74	2.70
	Adult	26.63	0.10	0.05	2.50	2.45	3.00	2.90

3ppm	I instar	8.48	0.05	0.03	0.80	0.77	1.03	1.00
	II instar	13.08	0.10	0.05	1.32	1.30	1.43	1.40
	III instar	40.08	0.10	0.05	1.32	1.30	1.43	1.40
	Prepupa	35.53	0.02	0.01	2.50	2.40	2.71	2.70
	Pupa	28.42	0.02	0.01	2.50	2.43	2.73	2.71
	Adult	23.18	0.13	0.06	2.40	2.36	2.54	2.50

4ppm	I instar	8.15	0.13	0.06	0.70	0.67	0.93	0.90
	II instar	12.10	0.14	0.07	1.22	1.22	1.35	1.31
	III instar	36.45	0.13	0.06	1.80	1.75	2.35	2.20
	Prepupa	33.33	0.24	0.12	2.35	2.32	2.57	2.52
	Pupa	26.63	0.15	0.08	2.30	2.25	2.43	2.42
	Adult	20.50	0.08	0.04	2.13	2.11	2.23	2.20

The weight (mg) of treated cultures is lower than that of untreated cultures, and it decreases as the concentration of lorazepam increases. Dev stage = development stage; SD = standard deviation; SE = standard error; min = minimum; CI = confidence interval; low = lower bound; up = upper bound; max = maximum.

## Data Availability

The data and the results used to support the findings of this study are uploaded to OSF and can be assessed using the given link (https://osf.io/zd269/?view_only=d3081187a13040d5b04c26048901f1c4).

## Supplementary Materials

Supplementary Materials Supplementary data consist of Statistical analysis performed using SPSS and one-way ANOVA with post hoc Tukey's test performed for comparisons and all the observations. Developmental time of the treated and untreated groups was compared, and estimated mean and standard deviation with standard errors are presented in the table. Similarly, morphometric parameters such length, width, and weight were also compared and shown in the supplementary file. ANOVA and Tukey's comparison results show significance level to *α* = 0.05.
