# Supplementary material for: Effect of Lorazepam on the Development of the Hairy Maggot Blow Fly, Chrysomya rufifacies (Macquart): Implication for Forensic Entomology
Source: J Toxicol. 2023 Jul 10;2023:1051736. doi: 10.1155/2023/1051736 (PMC11074883; doi:10.1155/2023/1051736)
Supplement: Supplementary Materials — Supplementary data consist of Statistical analysis performed using SPSS and one-way ANOVA with post hoc Tukey's test performed for comparisons and all the observations. Developmental time of the treated and untreated groups was compared, and estimated mean and standard deviation with standard errors are presented in the table. Similarly, morphometric parameters such length, width, and weight were also compared and shown in the supplementary file. ANOVA and Tukey's comparison results show significance level to α = 0.05. [file 1051736.f1.docx]

**Table 1**: Effect of Lorazepam on the duration of developmental stages of control and treated cultures of *C. megacephala*

| **Development stages** | **Doses** | **Mean** | **Std. Deviation** | **Std. Error** |
| --- | --- | --- | --- | --- |
| **I_instar_D** | Control | 22.5875 | 0.57209 | 0.28605 |
|  | 1 ppm | 24.4625 | 0.48883 | 0.24442 |
|  | 2ppm | 27.1750 | 0.77082 | 0.38541 |
|  | 3ppm | 32.9625 | 0.37722 | 0.18861 |
|  | 4ppm | 35.3500 | 0.50662 | 0.25331 |
| **II_instar_D** | Control | 24.3125 | 0.52974 | 0.26487 |
|  | 1 ppm | 26.8625 | 0.44605 | 0.22302 |
|  | 2ppm | 29.9625 | 0.37722 | 0.18861 |
|  | 3ppm | 33.6125 | 0.50724 | 0.25362 |
|  | 4ppm | 35.6125 | 0.50724 | 0.25362 |
| **III_instar_D** | Control | 28.1750 | 0.66521 | 0.3326 |
|  | 1 ppm | 29.9000 | 0.62183 | 0.31091 |
|  | 2ppm | 32.2250 | 0.6994 | 0.3497 |
|  | 3ppm | 34.9250 | 0.3594 | 0.1797 |
|  | 4ppm | 39.6000 | 0.52281 | 0.26141 |
| **prepupa_D** | Control | 33.4875 | 0.35678 | 0.17839 |
|  | 1 ppm | 37.7000 | 0.40825 | 0.20412 |
|  | 2ppm | 42.2125 | 0.70519 | 0.3525 |
|  | 3ppm | 54.5750 | 0.39686 | 0.19843 |
|  | 4ppm | 62.3625 | 0.42696 | 0.21348 |
| **pupa_D** | Control | 148.7000 | 0.66833 | 0.33417 |
|  | 1 ppm | 153.6375 | 0.48197 | 0.24098 |
|  | 2ppm | 157.6625 | 0.55883 | 0.27942 |
|  | 3ppm | 168.0250 | 0.45735 | 0.22867 |
|  | 4ppm | 177.8000 | 0.46904 | 0.23452 |
| **adult_D** | Control | 257.2625 | 1.85174 | 0.92587 |
|  | 1 ppm | 272.5625 | 1.47556 | 0.73778 |
|  | 2ppm | 289.2375 | 1.15208 | 0.57604 |
|  | 3ppm | 324.1000 | 0.44907 | 0.22454 |
|  | 4ppm | 350.7250 | 1.00374 | 0.50187 |
| **Table 2: Effect of Lorazepam on the length of developmental stages of *C. megacephala* in control and treated cultures** | | | | |
| **Development stages** | **Doses** | **Mean** | **Std. Deviation** | **Std. Error** |
| **I_instar_L** | Control | 4.2000 | 0.08165 | 0.04082 |
|  | 1 ppm | 3.9750 | 0.09574 | 0.04787 |
|  | 2ppm | 3.8000 | 0.08165 | 0.04082 |
|  | 3ppm | 3.3500 | 0.05774 | 0.02887 |
|  | 4ppm | 3.0500 | 0.05774 | 0.02887 |
| **II_instar_L** | Control | 8.1000 | 0.08165 | 0.04082 |
|  | 1 ppm | 7.1000 | 0.08165 | 0.04082 |
|  | 2ppm | 6.1250 | 0.09574 | 0.04787 |
|  | 3ppm | 5.7250 | 0.09574 | 0.04787 |
|  | 4ppm | 5.0750 | 0.09574 | 0.04787 |
| **III_instar_L** | Control | 16.3000 | 0.08165 | 0.04082 |
|  | 1 ppm | 14.3750 | 0.05 | 0.025 |
|  | 2ppm | 13.1250 | 0.09574 | 0.04787 |
|  | 3ppm | 11.0250 | 0.05 | 0.025 |
|  | 4ppm | 10.0250 | 0.05 | 0.025 |
| **Prepupa_L** | Control | 13.5000 | 0.08165 | 0.04082 |
|  | 1 ppm | 11.5750 | 0.0866 | 0.0433 |
|  | 2ppm | 10.5000 | 0.08165 | 0.04082 |
|  | 3ppm | 9.9250 | 0.02887 | 0.01443 |
|  | 4ppm | 8.8750 | 0.05 | 0.025 |
| **Pupa_L** | Control | 10.4250 | 0.09574 | 0.04787 |
|  | 1 ppm | 9.4250 | 0.09574 | 0.04787 |
|  | 2ppm | 9.0500 | 0.05774 | 0.02887 |
|  | 3ppm | 8.4500 | 0.05774 | 0.02887 |
|  | 4ppm | 7.4750 | 0.05 | 0.025 |
| **Adult_L** | Control | 10.2750 | 0.05 | 0.025 |
|  | 1 ppm | 9.0750 | 0.09574 | 0.04787 |
|  | 2ppm | 8.5000 | 0.08165 | 0.04082 |
|  | 3ppm | 8.0750 | 0.05 | 0.025 |
|  | 4ppm | 7.1750 | 0.05 | 0.025 |

**Table 3**: Effect of Lorazepam on the weight of *C. megacephala* in control and treated cultures

| **Development stages** | **Doses** | **Mean** | **Std. Deviation** | **Std. Error** |
| --- | --- | --- | --- | --- |
| **I_instar_W** | Control | 9.45 | 0.05774 | 0.02887 |
|  | 1 ppm | 9.125 | 0.15 | 0.075 |
|  | 2ppm | 8.6 | 0.11547 | 0.05774 |
|  | 3ppm | 8.475 | 0.05 | 0.025 |
|  | 4ppm | 8.15 | 0.1291 | 0.06455 |
| **II_instar_W** | Control | 19.155 | 0.12923 | 0.06461 |
|  | 1 ppm | 16.2025 | 0.14151 | 0.07075 |
|  | 2ppm | 14.575 | 0.09574 | 0.04787 |
|  | 3ppm | 13.075 | 0.09574 | 0.04787 |
|  | 4ppm | 12.1 | 0.14142 | 0.07071 |
| **III_instar_W** | Control | 51.25 | 0.17321 | 0.0866 |
|  | 1 ppm | 46.55 | 0.05774 | 0.02887 |
|  | 2ppm | 43.125 | 0.15 | 0.075 |
|  | 3ppm | 40.075 | 0.09574 | 0.04787 |
|  | 4ppm | 36.45 | 0.1291 | 0.06455 |
| **Prepupa_W** | Control | 46.775 | 0.1893 | 0.09465 |
|  | 1 ppm | 41.855 | 0.00577 | 0.00289 |
|  | 2ppm | 40.675 | 0.20616 | 0.10308 |
|  | 3ppm | 35.5325 | 0.02217 | 0.01109 |
|  | 4ppm | 33.325 | 0.23629 | 0.11815 |
| **Pupa_W** | Control | 40.7125 | 0.01258 | 0.00629 |
|  | 1 ppm | 34.4125 | 0.00957 | 0.00479 |
|  | 2ppm | 31.89 | 0.02708 | 0.01354 |
|  | 3ppm | 28.415 | 0.01732 | 0.00866 |
|  | 4ppm | 26.625 | 0.15 | 0.075 |
| **Adult_W** | Control | 37.55 | 0.1 | 0.05 |
|  | 1 ppm | 30.3825 | 0.08808 | 0.04404 |
|  | 2ppm | 26.625 | 0.09574 | 0.04787 |
|  | 3ppm | 23.175 | 0.12583 | 0.06292 |
|  | 4ppm | 20.5 | 0.08165 | 0.04082 |
|  |  |  |  |  |
| **Development stages** | **Doses** | **Mean** | **Std. Deviation** | **Std. Error** |
| **I_instar_Wd** | Control | 1.4750 | .09574 | .04787 |
|  | 1 ppm | 1.3500 | .05000 | .02500 |
|  | 2ppm | 1.1500 | .09574 | .04787 |
|  | 3ppm | .9000 | .04123 | .02062 |
|  | 4ppm | .8000 | .19149 | .09574 |
| **II_instar_Wd** | Control | 1.8500 | .08539 | .04270 |
|  | 1 ppm | 1.5750 | .02363 | .01181 |
|  | 2ppm | 1.5750 | .07500 | .03750 |
|  | 3ppm | 1.3650 | .09678 | .04839 |
|  | 4ppm | 1.2850 | .07937 | .03969 |
| **III_instar_Wd** | Control | 3.5750 | .05000 | .02500 |
|  | 1 ppm | 3.4250 | .08165 | .04082 |
|  | 2ppm | 1.9750 | .09798 | .04899 |
|  | 3ppm | 1.3650 | .09535 | .04768 |
|  | 4ppm | 2.0500 | .05560 | .02780 |
| **Prepupa_Wd** | Control | 3.5875 | .07762 | .03881 |
|  | 1 ppm | 3.2325 | .08165 | .04082 |
|  | 2ppm | 2.8625 | .17078 | .08539 |
|  | 3ppm | 2.5550 | .05774 | .02887 |
|  | 4ppm | 2.4450 | .03775 | .01887 |
| **Pupa_Wd** | Control | 3.3750 | .09574 | .04787 |
|  | 1 ppm | 3.1000 | .05000 | .02500 |
|  | 2ppm | 2.5800 | .09574 | .04787 |
|  | 3ppm | 2.5825 | .04123 | .02062 |
|  | 4ppm | 2.3375 | .19149 | .09574 |
| **Adult_Wd** | Control | 3.3025 | .08539 | .04270 |
|  | 1 ppm | 2.8000 | .02363 | .01181 |
|  | 2ppm | 2.7250 | .07500 | .03750 |
|  | 3ppm | 2.4500 | .09678 | .04839 |
|  | 4ppm | 2.1675 | .07937 | .03969 |

**Table 4**: Effect of Lorazepam on the width of *C. megacephala* in control and treated cultures

**Table 5:** Effect and temperature and humidity on the life cycle of *C. megacephala*

| **Temperature** | **Doses** | **Std. Deviation** | **Std. Error** | **Humidity** | **Doses** | **Std. Deviation** | **Std. Error** |
| --- | --- | --- | --- | --- | --- | --- | --- |
| **Temperature_max** | Control | 1.15 | 0.575 | **Humidity_max** | Control | 0.1 | 0.05 |
|  | 1 ppm | 0.28723 | 0.14361 |  | 1 ppm | 0.8165 | 0.40825 |
|  | 2ppm | 0.28868 | 0.14434 |  | 2ppm | 0.47871 | 0.23936 |
|  | 3ppm | 0.94296 | 0.47148 |  | 3ppm | 0.62915 | 0.31458 |
|  | 4ppm | 0.24495 | 0.12247 |  | 4ppm | 0.263 | 0.1315 |
| **Temperature_min** | Control | 0.3594 | 0.1797 | **Humidity_min** | Control | 1 | 0.5 |
|  | 1 ppm | 0.38297 | 0.19149 |  | 1 ppm | 1.10868 | 0.55434 |
|  | 2ppm | 0.33665 | 0.16833 |  | 2ppm | 0.47871 | 0.23936 |
|  | 3ppm | 0.15 | 0.075 |  | 3ppm | 0.50662 | 0.25331 |
|  | 4ppm | 0.66583 | 0.33292 |  | 4ppm | 0.263 | 0.1315 |

Table 5 shows overall temperature (C^0^) and humidity (%) recorded during the experiment.

**Table: 6** Post hoc tests (Tukey) showing multiple comparison width of control and treated cultures

| **Dependent Variable (I Group)** | **(J) Group** | **Mean Difference (I-J)** | **Std. Error** | **Sig.** |
| --- | --- | --- | --- | --- |
| I_instar_Wd | control-1ppm | 0.125 | 0.04743 | 0.113 |
|  | control-2ppm | .32500* | 0.04743 | 0 |
|  | control-3ppm | .57500* | 0.04743 | 0 |
|  | control-4ppm | .67500* | 0.04743 | 0 |
|  | 1ppm-2ppm | .20000* | 0.04743 | 0.006 |
|  | 1ppm-3ppm | .45000* | 0.04743 | 0 |
|  | 1ppm-4ppm | .55000* | 0.04743 | 0 |
|  | 2ppm-3ppm | .25000* | 0.04743 | 0.001 |
|  | 2ppm-4ppm | .35000* | 0.04743 | 0 |
|  | 3ppm-4ppm | 0.1 | 0.04743 | 0.267 |
| II_instar_Wd | control-1ppm | .27500* | 0.05027 | 0.001 |
|  | control-2ppm | .27500* | 0.05027 | 0.001 |
|  | control-3ppm | .48500* | 0.05027 | 0 |
|  | control-4ppm | .56500* | 0.05027 | 0 |
|  | 1ppm-2ppm | 0 | 0.05027 | 1 |
|  | 1ppm-3ppm | .21000* | 0.05027 | 0.006 |
|  | 1ppm-4ppm | .29000* | 0.05027 | 0 |
|  | 2ppm-3ppm | .21000* | 0.05027 | 0.006 |
|  | 2ppm-4ppm | .29000* | 0.05027 | 0 |
|  | 3ppm-4ppm | 0.08 | 0.05027 | 0.524 |
| III_instar_Wd | control-1ppm | 0.15 | 0.07694 | 0.335 |
|  | control-2ppm | 1.60000* | 0.07694 | 0 |
|  | control-3ppm | 2.21000* | 0.07694 | 0 |
|  | control-4ppm | 1.52500* | 0.07694 | 0 |
|  | 1ppm-2ppm | 1.45000* | 0.07694 | 0 |
|  | 1ppm-3ppm | 2.06000* | 0.07694 | 0 |
|  | 1ppm-4ppm | 1.37500* | 0.07694 | 0 |
|  | 2ppm-3ppm | .61000* | 0.07694 | 0 |
|  | 2ppm-4ppm | -0.075 | 0.07694 | 0.862 |
|  | 3ppm-4ppm | -.68500* | 0.07694 | 0 |
| Prepupa_instar_Wd | control-1ppm | .35500* | 0.05398 | 0 |
|  | control-2ppm | .72500* | 0.05398 | 0 |
|  | control-3ppm | 1.03250* | 0.05398 | 0 |
|  | control-4ppm | 1.14250* | 0.05398 | 0 |
|  | 1ppm-2ppm | .37000* | 0.05398 | 0 |
|  | 1ppm-3ppm | .67750* | 0.05398 | 0 |
|  | 1ppm-4ppm | .78750* | 0.05398 | 0 |
|  | 2ppm-3ppm | .30750* | 0.05398 | 0 |
|  | 2ppm-4ppm | .41750* | 0.05398 | 0 |
|  | 3ppm-4ppm | 0.11 | 0.05398 | 0.296 |
| Pupa_instar_Wd | control-1ppm | .27500* | 0.05563 | 0.001 |
|  | control-2ppm | .79500* | 0.05563 | 0 |
|  | control-3ppm | .79250* | 0.05563 | 0 |
|  | control-4ppm | 1.03750* | 0.05563 | 0 |
|  | 1ppm-2ppm | .52000* | 0.05563 | 0 |
|  | 1ppm-3ppm | .51750* | 0.05563 | 0 |
|  | 1ppm-4ppm | .76250* | 0.05563 | 0 |
|  | 2ppm-3ppm | -0.0025 | 0.05563 | 1 |
|  | 2ppm-4ppm | .24250* | 0.05563 | 0.004 |
|  | 3ppm-4ppm | .24500* | 0.05563 | 0.004 |
| Adult_instar_Wd | control-1ppm | .50250* | 0.06828 | 0 |
|  | control-2ppm | .57750* | 0.06828 | 0 |
|  | control-3ppm | .85250* | 0.06828 | 0 |
|  | control-4ppm | 1.13500* | 0.06828 | 0 |
|  | 1ppm-2ppm | 0.075 | 0.06828 | 0.805 |
|  | 1ppm-3ppm | .35000* | 0.06828 | 0.001 |
|  | 1ppm-4ppm | .63250* | 0.06828 | 0 |
|  | 2ppm-3ppm | .27500* | 0.06828 | 0.008 |
|  | 2ppm-4ppm | .55750* | 0.06828 | 0 |
|  | 3ppm-4ppm | .28250* | 0.06828 | 0.007 |

Table: 7 Post hoc tests (Tukey) showing multiple comparison weight of control and treated cultures

| **Dependent Variable (I Group)** | **(J) Group** | **Mean Difference (I-J)** | **Std. Error** | **Sig.** |
| --- | --- | --- | --- | --- |
| I_instar_W | control-1ppm | .32500* | 0.07638 | 0.005 |
|  | control-2ppm | .85000* | 0.07638 | 0 |
|  | control-3ppm | .97500* | 0.07638 | 0 |
|  | control-4ppm | 1.30000* | 0.07638 | 0 |
|  | 1ppm-2ppm | .52500* | 0.07638 | 0 |
|  | 1ppm-3ppm | .65000* | 0.07638 | 0 |
|  | 1ppm-4ppm | .97500* | 0.07638 | 0 |
|  | 2ppm-3ppm | 0.125 | 0.07638 | 0.498 |
|  | 2ppm-4ppm | .45000* | 0.07638 | 0 |
|  | 3ppm-4ppm | .32500* | 0.07638 | 0.005 |
| II_instar_W | control-1ppm | 2.95250* | 0.08664 | 0 |
|  | control-2ppm | 4.58000* | 0.08664 | 0 |
|  | control-3ppm | 6.08000* | 0.08664 | 0 |
|  | control-4ppm | 7.05500* | 0.08664 | 0 |
|  | 1ppm-2ppm | 1.62750* | 0.08664 | 0 |
|  | 1ppm-3ppm | 3.12750* | 0.08664 | 0 |
|  | 1ppm-4ppm | 4.10250* | 0.08664 | 0 |
|  | 2ppm-3ppm | 1.50000* | 0.08664 | 0 |
|  | 2ppm-4ppm | 2.47500* | 0.08664 | 0 |
|  | 3ppm-4ppm | .97500* | 0.08664 | 0 |
| III_instar_W | control-1ppm | 4.70000* | 0.09037 | 0 |
|  | control-2ppm | 8.12500* | 0.09037 | 0 |
|  | control-3ppm | 11.17500* | 0.09037 | 0 |
|  | control-4ppm | 14.80000* | 0.09037 | 0 |
|  | 1ppm-2ppm | 3.42500* | 0.09037 | 0 |
|  | 1ppm-3ppm | 6.47500* | 0.09037 | 0 |
|  | 1ppm-4ppm | 10.10000* | 0.09037 | 0 |
|  | 2ppm-3ppm | 3.05000* | 0.09037 | 0 |
|  | 2ppm-4ppm | 6.67500* | 0.09037 | 0 |
|  | 3ppm-4ppm | 3.62500* | 0.09037 | 0 |
| Prepupa_instar_W | control-1ppm | 4.92000* | 0.11606 | 0 |
|  | control-2ppm | 6.10000* | 0.11606 | 0 |
|  | control-3ppm | 11.24250* | 0.11606 | 0 |
|  | control-4ppm | 13.45000* | 0.11606 | 0 |
|  | 1ppm-2ppm | 1.18000* | 0.11606 | 0 |
|  | 1ppm-3ppm | 6.32250* | 0.11606 | 0 |
|  | 1ppm-4ppm | 8.53000* | 0.11606 | 0 |
|  | 2ppm-3ppm | 5.14250* | 0.11606 | 0 |
|  | 2ppm-4ppm | 7.35000* | 0.11606 | 0 |
|  | 3ppm-4ppm | 2.20750* | 0.11606 | 0 |
| Pupa_instar_W | control-1ppm | 6.30000* | 0.04877 | 0 |
|  | control-2ppm | 8.82250* | 0.04877 | 0 |
|  | control-3ppm | 12.29750* | 0.04877 | 0 |
|  | control-4ppm | 14.08750* | 0.04877 | 0 |
|  | 1ppm-2ppm | 2.52250* | 0.04877 | 0 |
|  | 1ppm-3ppm | 5.99750* | 0.04877 | 0 |
|  | 1ppm-4ppm | 7.78750* | 0.04877 | 0 |
|  | 2ppm-3ppm | 3.47500* | 0.04877 | 0 |
|  | 2ppm-4ppm | 5.26500* | 0.04877 | 0 |
|  | 3ppm-4ppm | 1.79000* | 0.04877 | 0 |
| Adult_instar_W | control-1ppm | 7.16750* | 0.0703 | 0 |
|  | control-2ppm | 10.92500* | 0.0703 | 0 |
|  | control-3ppm | 14.37500* | 0.0703 | 0 |
|  | control-4ppm | 17.05000* | 0.0703 | 0 |
|  | 1ppm-2ppm | 3.75750* | 0.0703 | 0 |
|  | 1ppm-3ppm | 7.20750* | 0.0703 | 0 |
|  | 1ppm-4ppm | 9.88250* | 0.0703 | 0 |
|  | 2ppm-3ppm | 3.45000* | 0.0703 | 0 |
|  | 2ppm-4ppm | 6.12500* | 0.0703 | 0 |
|  | 3ppm-4ppm | 2.67500* | 0.0703 | 0 |

Table: 8 Post hoc tests (Tukey) showing multiple comparison length of control and treated cultures

| Dependent Variable (I Group) | (J) Group | Mean Difference (I-J) | Std. Error | Sig. |
| --- | --- | --- | --- | --- |
| I_instar_L | control-1ppm | .22500* | 0.05401 | 0.006 |
|  | control-2ppm | .40000* | 0.05401 | 0 |
|  | control-3ppm | .85000* | 0.05401 | 0 |
|  | control-4ppm | 1.15000* | 0.05401 | 0 |
|  | 1ppm-2ppm | .17500* | 0.05401 | 0.038 |
|  | 1ppm-3ppm | .62500* | 0.05401 | 0 |
|  | 1ppm-4ppm | .92500* | 0.05401 | 0 |
|  | 2ppm-3ppm | .45000* | 0.05401 | 0 |
|  | 2ppm-4ppm | .75000* | 0.05401 | 0 |
|  | 3ppm-4ppm | .30000* | 0.05401 | 0 |
| II_instar_L | control-1ppm | 1.00000* | 0.0639 | 0 |
|  | control-2ppm | 1.97500* | 0.0639 | 0 |
|  | control-3ppm | 2.37500* | 0.0639 | 0 |
|  | control-4ppm | 3.02500* | 0.0639 | 0 |
|  | 1ppm-2ppm | .97500* | 0.0639 | 0 |
|  | 1ppm-3ppm | 1.37500* | 0.0639 | 0 |
|  | 1ppm-4ppm | 2.02500* | 0.0639 | 0 |
|  | 2ppm-3ppm | .40000* | 0.0639 | 0 |
|  | 2ppm-4ppm | 1.05000* | 0.0639 | 0 |
|  | 3ppm-4ppm | .65000* | 0.0639 | 0 |
| III_instar_L | control-1ppm | 1.92500* | 0.0483 | 0 |
|  | control-2ppm | 3.17500* | 0.0483 | 0 |
|  | control-3ppm | 5.27500* | 0.0483 | 0 |
|  | control-4ppm | 6.27500* | 0.0483 | 0 |
|  | 1ppm-2ppm | 1.25000* | 0.0483 | 0 |
|  | 1ppm-3ppm | 3.35000* | 0.0483 | 0 |
|  | 1ppm-4ppm | 4.35000* | 0.0483 | 0 |
|  | 2ppm-3ppm | 2.10000* | 0.0483 | 0 |
|  | 2ppm-4ppm | 3.10000* | 0.0483 | 0 |
|  | 3ppm-4ppm | 1.00000* | 0.0483 | 0 |
| Prepupa_instar_L | control-1ppm | 1.92500* | 0.04916 | 0 |
|  | control-2ppm | 3.00000* | 0.04916 | 0 |
|  | control-3ppm | 3.57500* | 0.04916 | 0 |
|  | control-4ppm | 4.62500* | 0.04916 | 0 |
|  | 1ppm-2ppm | 1.07500* | 0.04916 | 0 |
|  | 1ppm-3ppm | 1.65000* | 0.04916 | 0 |
|  | 1ppm-4ppm | 2.70000* | 0.04916 | 0 |
|  | 2ppm-3ppm | .57500* | 0.04916 | 0 |
|  | 2ppm-4ppm | 1.62500* | 0.04916 | 0 |
|  | 3ppm-4ppm | 1.05000* | 0.04916 | 0 |
| Pupa_instar_L | control-1ppm | 1.00000* | 0.05244 | 0 |
|  | control-2ppm | 1.37500* | 0.05244 | 0 |
|  | control-3ppm | 1.97500* | 0.05244 | 0 |
|  | control-4ppm | 2.95000* | 0.05244 | 0 |
|  | 1ppm-2ppm | .37500* | 0.05244 | 0 |
|  | 1ppm-3ppm | .97500* | 0.05244 | 0 |
|  | 1ppm-4ppm | 1.95000* | 0.05244 | 0 |
|  | 2ppm-3ppm | .60000* | 0.05244 | 0 |
|  | 2ppm-4ppm | 1.57500* | 0.05244 | 0 |
|  | 3ppm-4ppm | .97500* | 0.05244 | 0 |
| Adult_instar_L | control-1ppm | 1.20000* | 0.0483 | 0 |
|  | control-2ppm | 1.77500* | 0.0483 | 0 |
|  | control-3ppm | 2.20000* | 0.0483 | 0 |
|  | control-4ppm | 3.10000* | 0.0483 | 0 |
|  | 1ppm-2ppm | .57500* | 0.0483 | 0 |
|  | 1ppm-3ppm | 1.00000* | 0.0483 | 0 |
|  | 1ppm-4ppm | 1.90000* | 0.0483 | 0 |
|  | 2ppm-3ppm | .42500* | 0.0483 | 0 |
|  | 2ppm-4ppm | 1.32500* | 0.0483 | 0 |
|  | 3ppm-4ppm | .90000* | 0.0483 | 0 |

Table: 9 Post hoc tests (Tukey) showing multiple comparison of

Development time of control and treated cultures

| Dependent Variable (I Group) | (J) Group | Mean Difference (I-J) | Std. Error | Sig. |
| --- | --- | --- | --- | --- |
| I_instar_D | control-1ppm | -1.87500* | 0.39489 | 0.002 |
|  | control-2ppm | -4.58750* | 0.39489 | 0 |
|  | control-3ppm | -10.37500* | 0.39489 | 0 |
|  | control-4ppm | -12.76250* | 0.39489 | 0 |
|  | 1ppm-2ppm | -2.71250* | 0.39489 | 0 |
|  | 1ppm-3ppm | -8.50000* | 0.39489 | 0 |
|  | 1ppm-4ppm | -10.88750* | 0.39489 | 0 |
|  | 2ppm-3ppm | -5.78750* | 0.39489 | 0 |
|  | 2ppm-4ppm | -8.17500* | 0.39489 | 0 |
|  | 3ppm-4ppm | -2.38750* | 0.39489 | 0 |
| II_instar_D | control-1ppm | -2.55000* | 0.33711 | 0 |
|  | control-2ppm | -5.65000* | 0.33711 | 0 |
|  | control-3ppm | -9.30000* | 0.33711 | 0 |
|  | control-4ppm | -11.30000* | 0.33711 | 0 |
|  | 1ppm-2ppm | -3.10000* | 0.33711 | 0 |
|  | 1ppm-3ppm | -6.75000* | 0.33711 | 0 |
|  | 1ppm-4ppm | -8.75000* | 0.33711 | 0 |
|  | 2ppm-3ppm | -3.65000* | 0.33711 | 0 |
|  | 2ppm-4ppm | -5.65000* | 0.33711 | 0 |
|  | 3ppm-4ppm | -2.00000* | 0.33711 | 0 |
| III_instar_D | control-1ppm | -1.72500* | 0.41483 | 0.006 |
|  | control-2ppm | -4.05000* | 0.41483 | 0 |
|  | control-3ppm | -6.75000* | 0.41483 | 0 |
|  | control-4ppm | -11.42500* | 0.41483 | 0 |
|  | 1ppm-2ppm | -2.32500* | 0.41483 | 0 |
|  | 1ppm-3ppm | -5.02500* | 0.41483 | 0 |
|  | 1ppm-4ppm | -9.70000* | 0.41483 | 0 |
|  | 2ppm-3ppm | 2.70000* | 0.41483 | 0 |
|  | 2ppm-4ppm | -4.67500* | 0.41483 | 0 |
|  | 3ppm-4ppm | -2.00000* | 0.33711 | 0 |
| Prepupa_D | control-1ppm | -4.21250* | 0.33631 | 0 |
|  | control-2ppm | -8.72500* | 0.33631 | 0 |
|  | control-3ppm | -21.08750* | 0.33631 | 0 |
|  | control-4ppm | -28.87500* | 0.33631 | 0 |
|  | 1ppm-2ppm | -4.51250* | 0.33631 | 0 |
|  | 1ppm-3ppm | -16.87500* | 0.33631 | 0 |
|  | 1ppm-4ppm | -24.66250* | 0.33631 | 0 |
|  | 2ppm-3ppm | -2.70000* | 0.41483 | 0 |
|  | 2ppm-4ppm | -20.15000* | 0.33631 | 0 |
|  | 3ppm-4ppm | -7.78750* | 0.33631 | 0 |
| Pupa_D | control-1ppm | -4.93750* | 0.37688 | 0 |
|  | control-2ppm | -8.96250* | 0.37688 | 0 |
|  | control-3ppm | -19.32500* | 0.37688 | 0 |
|  | control-4ppm | -29.10000* | 0.37688 | 0 |
|  | 1ppm-2ppm | -4.02500* | 0.37688 | 0 |
|  | 1ppm-3ppm | -14.38750* | 0.37688 | 0 |
|  | 1ppm-4ppm | -24.16250* | 0.37688 | 0 |
|  | 2ppm-3ppm | -10.36250* | 0.37688 | 0 |
|  | 2ppm-4ppm | -20.13750* | 0.37688 | 0 |
|  | 3ppm-4ppm | -9.77500* | 0.37688 | 0 |
| Adult_D | control-1ppm | -15.30000* | 0.90237 | 0 |
|  | control-2ppm | -31.97500* | 0.90237 | 0 |
|  | control-3ppm | -66.83750* | 0.90237 | 0 |
|  | control-4ppm | -93.46250* | 0.90237 | 0 |
|  | 1ppm-2ppm | -4.02500* | 0.37688 | 0 |
|  | 1ppm-3ppm | -14.38750* | 0.37688 | 0 |
|  | 1ppm-4ppm | -24.16250* | 0.37688 | 0 |
|  | 2ppm-3ppm | -34.86250* | 0.90237 | 0 |
|  | 2ppm-4ppm | -61.48750* | 0.90237 | 0 |
|  | 3ppm-4ppm | -26.62500* | 0.90237 | 0 |

Table 10: Anova results showing comparison between the group and within the groups

| ANOVA |  |  |  |  |  |  |
| --- | --- | --- | --- | --- | --- | --- |
|  |  | Sum of Squares | df | Mean Square | F | Sig. |
| I_instar_D | Between Groups | 479.403 | 4 | 119.851 | 384.291 | 0 |
|  | Within Groups | 4.678 | 15 | 0.312 |  |  |
| II_instar_D | Between Groups | 346.868 | 4 | 86.717 | 381.523 | 0 |
|  | Within Groups | 3.409 | 15 | 0.227 |  |  |
| III_instar_D | Between Groups | 323.003 | 4 | 80.751 | 234.627 | 0 |
|  | Within Groups | 5.162 | 15 | 0.344 |  |  |
| prepupa_D | Between Groups | 2324.148 | 4 | 581.037 | 2568.593 | 0 |
|  | Within Groups | 3.393 | 15 | 0.226 |  |  |
| pupa_D | Between Groups | 2192.359 | 4 | 548.09 | 1929.328 | 0 |
|  | Within Groups | 4.261 | 15 | 0.284 |  |  |
| adult_D | Between Groups | 23366.019 | 4 | 5841.505 | 3586.954 | 0 |
|  | Within Groups | 24.428 | 15 | 1.629 |  |  |
| I_instar_L | Between Groups | 3.51 | 4 | 0.877 | 150.429 | 0 |
|  | Within Groups | 0.087 | 15 | 0.006 |  |  |
| II_instar_L | Between Groups | 22.655 | 4 | 5.664 | 693.52 | 0 |
|  | Within Groups | 0.122 | 15 | 0.008 |  |  |
| III_instar_L | Between Groups | 102.172 | 4 | 25.543 | 5473.5 | 0 |
|  | Within Groups | 0.07 | 15 | 0.005 |  |  |
| Prepupa_L | Between Groups | 49.695 | 4 | 12.424 | 2570.431 | 0 |
|  | Within Groups | 0.072 | 15 | 0.005 |  |  |
| Pupa_L | Between Groups | 19.343 | 4 | 4.836 | 879.227 | 0 |
|  | Within Groups | 0.082 | 15 | 0.005 |  |  |
| Adult_L | Between Groups | 21.382 | 4 | 5.346 | 1145.464 | 0 |
|  | Within Groups | 0.07 | 15 | 0.005 |  |  |
| I_instar_W | Between Groups | 4.353 | 4 | 1.088 | 93.279 | 0 |
|  | Within Groups | 0.175 | 15 | 0.012 |  |  |
| II_instar_W | Between Groups | 124.016 | 4 | 31.004 | 2065.325 | 0 |
|  | Within Groups | 0.225 | 15 | 0.015 |  |  |
| III_instar_W | Between Groups | 523.753 | 4 | 130.938 | 8016.628 | 0 |
|  | Within Groups | 0.245 | 15 | 0.016 |  |  |
| Prepupa_W | Between Groups | 454.545 | 4 | 113.636 | 4218.382 | 0 |
|  | Within Groups | 0.404 | 15 | 0.027 |  |  |
| Pupa_W | Between Groups | 490.553 | 4 | 122.638 | 25782.374 | 0 |
|  | Within Groups | 0.071 | 15 | 0.005 |  |  |
| Adult_W | Between Groups | 710.701 | 4 | 177.675 | 17974.228 | 0 |
|  | Within Groups | 0.148 | 15 | 0.01 |  |  |
| I_instar_wd | Between Groups | 1.318 | 4 | 0.33 | 73.222 | 0 |
|  | Within Groups | 0.067 | 15 | 0.004 |  |  |
| II_instar_wd | Between Groups | 0.775 | 4 | 0.194 | 38.331 | 0 |
|  | Within Groups | 0.076 | 15 | 0.005 |  |  |
| III_instar_wd | Between Groups | 15.101 | 4 | 3.775 | 318.85 | 0 |
|  | Within Groups | 0.178 | 15 | 0.012 |  |  |
| Prepupa_wd | Between Groups | 3.616 | 4 | 0.904 | 155.106 | 0 |
|  | Within Groups | 0.087 | 15 | 0.006 |  |  |
| Pupa_wd | Between Groups | 2.92 | 4 | 0.73 | 117.95 | 0 |
|  | Within Groups | 0.093 | 15 | 0.006 |  |  |
| Adult_wd | Between Groups | 2.876 | 4 | 0.719 | 77.127 | 0 |
|  | Within Groups | 0.14 | 15 | 0.009 |  |  |
